# The Neecham Confusion Scale and the Delirium Observation Screening Scale: Capacity to discriminate and ease of use in clinical practice

**DOI:** 10.1186/1472-6955-6-3

**Published:** 2007-03-29

**Authors:** Liesbeth A Gemert van, Marieke J Schuurmans

**Affiliations:** 1 VU Medical Centre Amsterdam, De Boellelaan 1117, 1081 HV Amsterdam, the Netherlands; 2 Academic Medical Centre, PO Box 22660, Roomnumber F4-108, 1100 DD Amsterdam, the Netherlands; 3 University of Professional Education, Bolognalaan 101, 3584 CJ Utrecht, The Netherlands

## Abstract

**Background:**

Delirium is a frequent form of psychopathology in elderly hospitalized patients; it is a symptom of acute somatic illness. The consequences of delirium include high morbidity and mortality, lengthened hospital stay, and nursing home placement. Early recognition of delirium symptoms enables the underlying cause to be diagnosed and treated and can prevent negative outcomes. The aim of this study was to determine which of the two delirium observation screening scales, the NEECHAM Confusion Scale or the Delirium Observation Screening (DOS) scale, has the best discriminative capacity for diagnosing delirium and which is more practical for daily use by nurses.

**Methods:**

The project was conducted on four wards of a university hospital; 87 patients were included. During 3 shifts, these patients were observed for symptoms of delirium, which were rated on both scales. A DSM-IV diagnosis of delirium was made or rejected by a geriatrician. Nurses were asked to rate the practical value of both scales using a structured questionnaire.

**Results:**

The sensitivity (0.89 – 1.00) and specificity (0.86 – 0.88) of the DOS and the NEECHAM were high for both scales. Nurses rated the practical use of the DOS scale as significantly easier than the NEECHAM.

**Conclusion:**

Successful implementation of standardized observation depends largely on the consent of professionals and their acceptance of a scale. In our hospital, we therefore chose to involve nurses in the choice between two instruments. During the study they were able to experience both scales and give their opinion on ease of use. In the final decision on the instrument we found that both scales were very acceptable in terms of sensitivity and specificity, so the opinion of the nurses was decisive. They were positive about both instruments; however, they rated the DOS scale as significantly easier to use and relevant to their practice. Our findings were obtained from a single site study with a small sample, so a large comparative trial to study the value of both scales further is recommended. On the basis of our experience during this study and findings from the literature with regard to the implementation of delirium guidelines, we will monitor the further implementation of the DOS Scale in our hospital with intensive consultation.

## Background

Delirium is a frequent form of psychopathology in elderly hospitalized patients; it is a symptom of acute somatic illness. Serious conditions such as a heart attack may present in elderly patients with no symptoms other than delirium. The consequences of delirium include high morbidity and mortality, lengthened hospital stay, and nursing home placement [1]. Caring for a delirious patient is experienced as burdensome by nurses. Early recognition of delirium symptoms enables the underlying cause to be diagnosed and treated and can prevent negative outcomes [2]. The main symptoms of delirium are a disturbance of consciousness with reduced attention and a change in cognition or perceptual disturbances [3]. Symptoms develop in hours to days and fluctuate over the course of the day. Owing to the fluctuating nature and different presentations of the condition, delirium is difficult to diagnose and is often missed.

Nurses have frequent round-the-clock contacts with patients and are in a strategic position to observe changes in behaviour [4,5]. However, they are not well trained in recognizing delirium. In our experience, they observe behavioural changes in patients but often do not define them as symptoms of delirium. In VU university medical centre, a 733 bed university hospital, we therefore decided to introduce a standardised scale to enhance the recognition of delirium.

To implement delirium screening successfully, nurses need instruments that are based on observation and allow bedside use during regular care, repetitively and without respondent burden [6-8]. Two scales have been developed that meet these criteria: the NEECHAM Confusion Scale [9] and the Delirium Observation Screening (DOS) Scale [1]. Both scales have been developed to rate nurses' observations during regular care and have been tested on several samples with good results.

The NEECHAM Confusion Scale [9] was developed to assess acute confusion on the basis of criteria identified by nurses as representing acute confusion. The instrument has been tested on several samples [7-9] and shows good internal consistency (0.85 – 0.90), inter-rater reliability (0.91 – 0.96) and test-retest reliability (0.98). Validity has been evaluated by calculating the correlation with the Mini Mental State Examination (MMSE) (0.50 – 0.87), nurses' reports of confusion (0.43 – 0.46) and patients' self-reports of confusion (0.40 – 0.44). The correlation with a DSM-III-R diagnosis ranged from -0.54 to -0.70. Construct validity has been tested by correlation with several measures of functional status (0.47 – 0.70). Analysis of variance showed two components explaining 72% of all variance.

The Delirium Observation Screening Scale [11] was developed on the basis of the DSM-IV criteria for delirium and tested for content validity by a group of experts in the field of delirium. In two prospective studies with high-risk groups of patients, the DOS Scale showed high internal consistency (0.93 – 0.96) [11,12]. Predictive validity against the Diagnostic and Statistical Manual-IV diagnosis of delirium made by a geriatrician was good in both studies. Correlations of the DOS Scale with the MMSE were -0.66 and -0.79. Concurrent validity, as tested by comparison of the research nurse's ratings of the DOS Scale and the Confusion Assessment Method (CAM), was 0.63. Construct validity of the DOS has been tested against the Informant Questionnaire of Cognitive Decline in Elderly (IQCODE) (0.33 and 0.74) and the Barthel Index (-0.26 and -0.55). An algorithm of 13 items rated over 3 consecutive shifts has been developed. The sensitivity of this algorithm was 0.94, specificity 0.77 [12].

Both scales were developed for nurses without specific training in geriatric care. However, they have never been compared to each other in one study. This comparative study was designed in order to decide which instrument to implement in our hospital. The aim of the study was to test the discriminative value of each scale and to determine their ease of use in daily care.

## Methods

### Design and sample

The discriminative value of each scale was tested in a prospective study on high-risk patients. The study was conducted on a general medical and three surgical wards in a university hospital. Patients were included if they were aged seventy years or over, had three or more comorbid problems and were Dutch or English speaking. Since the goal of the study was to determine the discriminative value of standardised observations, patients who were already diagnosed as delirious were excluded. In those patients, the observations would not have been blind; nurses already knew the diagnosis and this would have guided their observations and their care. Of the 223 elderly patients admitted during the time of the study, 98 met the inclusion criteria. For organizational reasons, 11 were not included. The average age of the patients was 79 years (range 70–96 years); 52% were female.

The practical use of both scales was determined by the nurses on the participating wards. All nurses were asked to participate in the study. A total of 39 participated. Most nurses (88%) were trained at BsN level, their mean age was 34, and the mean number of years working as a nurse was 13.

The hospital's Medical Ethical Committee approved this study.

### Procedure

Patients admitted to the wards received written information about the study from the ward staff. They were asked to participate in the study and give their consent. If they were not able to consent, surrogate consent was requested from their families. During the five month study period, a clinical nurse specialist included patients on each ward on a fixed day every other week. For example, on the general medical ward, patients were included on Tuesday in the even weeks. A maximum of 4 patients were included per ward per study day. If there were more eligible patients on a certain day, they were selected in alphabetical order. This procedure was chosen to balance the workload for the study physician who visited all the patients. The patients included were observed by nurses for symptoms of delirium during three shifts: evening, night and day. At the end of each shift, for each patient included in the study, a nurse rated her observation of her patient on both scales. At the end of the day shift when all the nursing data had been collected, patients were seen by a geriatrician who made or rejected the DSM-IV diagnosis of delirium. The geriatrician was blinded to the nurses' ratings.

At the end of the study period, the nurses were asked to rate the practical value of both scales using a validated structured questionnaire [10]. The items in this questionnaire are given in table 4. This questionnaire evaluates the language used to describe the behavioural observations and the knowledge and time needed to rate the scale. Responses were given on a 4-point Likert scale.

**Table 4:** Ease of use of the DOS Scale and the NEECHAM Confusion Scale

Items	Agreement (%) DOS (N = 37)	Agreement (%) NEECHAM (N = 31)	p-value
How much time did you need to rate this scale	5 minutes	8 minutes	***0.003***
The concepts of the scale were clear to me	100%	96.7%	0.26
The concepts were compatible with the language used in practice	100%	83.9%	***0.009***
I have sufficient knowledge from my training/experience to evaluate the observations on the scale	100%	93.6%	0.135
The way in which the observations are described is free of values and judgement	97.3%	96.8%	0.826
The observations can be interpreted in various ways	24.3%	29.1%	0.440
There was a clear difference between the possible answers	89.2%	74.2%	***0.021***
I could quickly make a choice between the possible answers	100%	71%	***0.000***
The instructions on the form helped me in choosing the answers	74.6%	80.6%	0.542
I requested help from others because it was not clear to me what was being asked	2.7%	19.4%	***0.010***
I found it a handy instrument to spot delirium symptoms	91.9%	54.8%	***0.000***
This instrument offered added value to my practice of nursing	83.8%	54.8%	***0.004***

### The NEECHAM Confusion Scale

The NEECHAM Confusion Scale contains nine scaled items divided into three subscales (table 1). Each scaled item gives 3 descriptions. Subscale I, information processing (score range 0 – 14 points), evaluates components of cognitive status: attention and alertness, verbal and motor response, and memory and orientation. Subscale II, behaviour (score range 0 – 10 points), evaluates observed behaviour and performance ability: general appearance and posture, sensory-motor performance, and verbal responses. Subscale III, performance (score range 0 – 16 points), assesses vital function: vital signs, oxygen saturation level and urinary incontinence. The total NEECHAM scale score is the sum of the scores on the three levels. The scale can be rated in 10 minutes on the basis of observations and measurements of vital signs. The scores may range from zero (minimal function) to 30 (normal function); the cut-off point is 24. The range from 0–24 points indicates a delirium.

**Table 1:** NEECHAM Confusion Scale

Subscale I Level of responsiveness-information processing
• attention and alertness (0 – 4 points)
• verbal and motor response (0 – 5 points)
• memory and orientation (0 – 5 points)
Subscale II Level of behaviour
• general behaviour and posture (0 – 2 points)
• sensory motor performance (0 – 4 points)
• verbal responses (0 – 4 points)
Subscale III Vital functions
• vital signs (0 – 2 points)
• oxygen saturation level (0 – 2 points)
• urinary continence (0 – 2 points)
Scores: 0 – 19 points = moderate to severe confusion
20 – 24 points = mild or early development of delirium
25 – 30 points = not confused or normal function

### The Delirium Observation Screening Scale

At the development stage, the DOS Scale was designed with 25 behavioural items that were rated on a 5-point Likert scale [11]. On the basis of studies on geriatric and hip fracture patients, the scale was reduced on 13 items (table 2) that can be rated as present or absent in less than 5 minutes [12]. A score of 0 is defined as 'normal behaviour', meaning absence of behavioural alterations. Three items (3, 8 and 9) are reverse-scored, i.e. 'normal behaviour' is rated as 'always'. The highest total score is 13; the cut-off point is 3. Three or more points indicates a delirium.

**Table 2:** The DOS Scale

	The patient:
1	Dozes during conversation or activities
2	Is easy distracted by stimuli from the environment
3	Maintains attention to conversation or action
4	Does not finish question or answer
5	Gives answers which do not fit the question
6	Reacts slowly to instructions
7	Thinks to be somewhere else
8	Knows which part of the day it is
9	Remembers recent event
10	Is picking, disorderly, restless
11	Pulls IV tubes, feeding tubes, catheters etc.
12	Is easy or sudden emotional (frightened, angry, irritated)
13	Sees persons/things as somebody/something else

Never = 0 point; Sometimes or always = 1 point

Items 3, 8 and 9 are rated in reverse

### Statistical analysis

Data were analysed using SPSS. Chi square tests were used to calculate the sensitivity and specificity of the ratings on both scales as compared with the DSM-IV diagnoses. Chi square tests were also used to compare the ratings of the nurses with regard to ease of use of both scales.

## Results

Delirium was diagnosed in nine (10.3%) of the eighty-seven patients seen by the geriatrician. Sensitivity and specificity of both scales was determined (table 3). The total DOS Scale score was based on observations during three shifts. The NEECHAM ratings gave a total score based on a single rating. Therefore the NEECHAM ratings are given per shift and a mean score over three shifts is also calculated. Both scales showed high sensitivity and specificity. However, the positive predictive value is less than 50%, except for the NEECHAM day shift. The negative predictive value is high, almost 100% in all ratings.

For 15 of the 16 patients who were classified 'delirious false positive' by both instruments, the medical chart was studied with regard to known factors that may be confounded with delirium symptoms. Two of these patients became delirious later during admission. All 15 patients were living at home before admission, 7 alone, 8 with a partner. Eleven patients (73%) received more than 3 new medications during admission; 20% had no psychiatric comorbidity, 20% suffered from amnesia (amnesic syndrome/early dementia) and 60% had psychiatric comorbidity (dementia, depression, alcohol abuse, bipolar disorder).

Thirty-nine nurses completed the ease-of-use questionnaire; 37 evaluated the DOS and 31 evaluated the NEECHAM. Most nurses (88%) were trained at BsN level; their mean age was 34 and the mean number of years working as a nurse was 13.

The first question regarding ease of use concerned the time needed to rate the scale. The mean time needed to rate the NEECHAM was 8 minutes (range: 3–15 minutes, median 7 minutes). The mean time needed to rate the DOS was 5 minutes (range; 1–15 minutes, median 5 minutes). Rating the DOS scale took significantly less time (p < 0.003), but the DOS scale needed to be rated in three successive shifts in order to indicate a diagnosis. The results from the structured questionnaire are given in table 4.

**Table 3:** Sensitivity and specificity of DOS Scale and NEECHAM

	N	Sens	Spec	PV+	PV-
DOS scale, 3 shifts	86	0,89	0,88	47,0%	98,5%
NEECHAM, 3 shifts	68	1	0,87	43%	100%
NEECHAM evening shift	82	1	0,86	44,4%	100%
NEECHAM night shift	74	0,86	0,86	40,0%	98,3%
NEECHAM day shift	82	0,89	0,90	53,3%	98,5%

Nurses were positive about both instruments; however, they rated the DOS Scale as significantly easier to use and more relevant to their practice.

## Discussion

This study was undertaken to guide the choice for implementing a delirium-screening instrument in clinical practice. Data were collected on eighty-seven elderly hospitalized patients. The prevalence of delirium in this group was low compared to other studies on vulnerable elderly patients. This may be influenced by the facts that the study did not follow patients prospectively and that incident cases who had already been reported and diagnosed were excluded from the study. Ratings of the observation of behaviour of already-diagnosed patients would not have been reliable given our question about the discriminative value of both scales.

The results regarding the sensitivity and specificity of each scale were comparable with those from other studies. The NEECHAM scale had a sensitivity of 0.95 and a specificity of 0.78 in earlier studies, and a sensitivity range from 1–0.86 and a specificity range from 0.86 to 0.90 in this study. The DOS Scale had a sensitivity of 0.94 and a specificity of 0.77 in earlier studies and a sensitivity of 0.89 and a specificity of 0.88 in this study. The specificities of both scales were a little higher than in earlier reports.

Of the false-positive rated patients, 80% were vulnerable in their mental state. It is known that patients with psychiatric comorbidity, especially those with dementia, show behaviour that is sometimes hard to distinguish from delirium [13]. The NEECHAM Confusion Scale and the DOS Scale, however, both serve as screening tools and not as diagnostic tools. For a screening tool, sensitivity is more important than specificity. The fact that some vulnerable patients are false-positively rated will not interfere with the goal of screening, since further assessment of these patients by a nurse specialist will enhance their quality of care and may prevent a future delirium.

The study described here could be questioned with regard to the relatively small sample size and data collection during regular care. The sample size was influenced by the maximal duration of the study and the fact that for practical reasons we had to limit the number of patients on every round to four. If there were more eligible patients on a certain day, patients were selected in alphabetical order.

Observing behaviour is a difficult matter. Both scales were developed for nurses without specific training in the area of gerontology or behavioural alterations. In the busy situation in which nurses work, filling out forms can give problems. The strength of this method, however, lies in the value of the results for clinical practice. The results of this study confirm earlier findings that these instruments enable nurses to recognize a possible delirium in an early stage so that treatment of the underlying etiological factors is possible and the consequences of the disturbed behaviour can be prevented. Finally, the reliability of a diagnosis made by a physician as 'gold standard' can be questioned. In delirium research, however, there is no alternative standard. Strict use of the DSM criteria enhances reliability.

## Conclusion

Successful implementation of standardized observation depends largely on the consent of professionals and their acceptation of a scale. In our hospital, we therefore choose to involve nurses in the choice of two instruments. During the study they were able to experience both scales and give their opinion on ease of use. In the final decision on the instrument, we found that both scales were very acceptable in respect of sensitivity and specificity, so the opinion of the nurses was decisive. Nurses were positive about both instruments; however, they rated the DOS scale as significantly easier to use and relevant to their practice. Our findings result from a single site study with a small sample, so a large comparative trial to study the value of both scales further is recommended. On the basis of our experiences during this study and findings from the literature with regard to implementation of delirium guidelines, we will monitor the further implementation of the DOS Scale with intensive consultation.

## Competing interests

The authors declare that they have no competing interests. MS originally developed the DOS Scale. In her practice and research over the last years she has used both the NEECHAM Confusion Scale and the DOS Scale, depending on the patient population and on preferences of the nurses with whom she conducts the research. The focus in her research is to enhance recognition of delirium by nurses. She is also involved in studies with the Delirium Rating Scale and with the Confusion Assessment Method.

## Authors' contributions

MS proposed the original idea for the study based on a question raised in LG's clinical practice. LG and MS designed the study protocol. LG executed the study and the statistical analyses. MS was involved in the interpretation of data. LG drafted the manuscript. MS prepared the international publication. Both authors approved the manuscript.

## Pre-publication history

The pre-publication history for this paper can be accessed here:



## References

[B1] Schuurmans MJ (2001). Early recognition of delirium. PhD thesis.

[B2] Schuurmans MJ, Shortridge-Bagget LM, Duursma SA (2001). Early recognition of delirium: review of the literature. Journal of Clinical Nursing.

[B3] American Psychiatric Association (1999). Practice guidelines for the treatment of patients with delirium. The American Journal of Psychiatry.

[B4] Yeaw EM, Abbate JH (1993). Identification of confusion among the elderly in an acute care setting. Clinical Nurse Specialist.

[B5] Morency CR, Levkoff SE, Dick KL (1994). Research considerations delirium in hospitalized elders. Journal of Gerontological Nursing.

[B6] Williams MA, Ward SE, Campbell EB (1988). Confusion: testing versus observation. Journal of Gerontological Nursing.

[B7] Neelon VJ, Champagne MT, McConnel E, Carlson J, Funk SG (1992). Use of the NEECHAM Confusion Scale to assess acute confusional states of hospitalized older patients. Key aspects of elder care; managing falls, incontinence and cognitive impairment.

[B8] Neelon VJ, Champagne MT, Carlson JR, Funk SG (1996). The NEECHAM Confusion Scale: construction, validation, and clinical testing. Nursing Research.

[B9] Champagne MT, Neelon VJ, McConnel ES, Funk S (1987). The NEECHAM Confusion Scale: assessing acute confusion in hospitalized and nursing home elderly. The Gerontologist.

[B10] Roodbol G (1998). Detection of delirium by nurses at a general hospital using the VOVSD. PhD thesis.

[B11] Schuurmans MJ, Shortridge-Bagget LM, Duursma SA The Delirium Observation Screening Scale: a screening instrument for delirium. Research & Theory for Nursing Practices.

[B12] Schuurmans MJ, Donders RT, Shortridge-Bagget LM, Duursma SA (2002). Delirium case finding: pilot testing of a new screening scale for nurses. JAGS.

[B13] MacDonald (1999). Can delirium be separated from dementia?. Dement Geriatr Cogn Disord.

